# Overview on the Role of E-Cadherin in Gastric Cancer: Dysregulation and Clinical Implications

**DOI:** 10.3389/fmolb.2021.689139

**Published:** 2021-07-30

**Authors:** Huichen Zhao, Huihui Hu, Beibei Chen, Weifeng Xu, Jing Zhao, Chen Huang, Yishu Xing, Huifang Lv, Caiyun Nie, Jianzheng Wang, Yunduan He, Sai-Qi Wang, Xiao-Bing Chen

**Affiliations:** ^1^ Department of Medical Oncology, Affiliated Cancer Hospital of Zhengzhou University, Henan Cancer Hospital, Zhengzhou, China; ^2^ State Key Laboratory of Esophageal Cancer Prevention and Treatment, Zhengzhou University, Zhengzhou, China

**Keywords:** gastric cancer, e-cadherin, CDH1, EMT, precision therapy

## Abstract

Gastric cancer is the fifth most common cancer and the third most common cause of cancer death all over the world. E-cadherin encoded by human *CDH1* gene plays important roles in tumorigenesis as well as in tumor progression, invasion and metastasis. Full-length E-cadhrin tethered on the cell membrane mainly mediates adherens junctions between cells and is involved in maintaining the normal structure of epithelial tissues. After proteolysis, the extracellular fragment of the full-length E-cadhein is released into the extracellular environment and the blood, which is called soluble E-cadherin (sE-cadherin). sE-cadherin promots invasion and metastasis as a paracrine/autocrine signaling molecule in the progression of various types of cancer including gastric cancer. This review mainly summarizes the dysregulation of E-cadherin and the regulatory roles in the progression, invasion, metastasis, and drug-resistance, as well as its clinical applications in diagnosis, prognosis, and therapeutics of gastric cancer.

## Introduction

Gastric cancer (GC) is one of the common malignant tumors of the digestive tract. The incidence and mortality of gastric cancer are much higher than the world average, which seriously affects our health (Sung et al., 2021). And it is a multifactorial disease. Both genetic and environmental factors are important to its pathogenesis. Gastric cancer is mostly sporadic, but about 10% of gastric cancers have family clustering characteristics, of which 1/3 are considered to have genetic background. This part of gastric cancer is called hereditary diffuse gastric cancer (HDGC) (Blair et al., 2020). In addition, smoking (Shikata et al., 2008) and alcohol consumption (Deng et al., 2021), eating habits (Takezaki et al., 1999; Kobayashi et al., 2002), chronic atrophic gastritis, and EBV or *Helicobacter pylori* infection (Arif and Syed, 2007; Holleczek et al., 2020) have been considered the most important risk factors for gastric cancer (FORMAN, 1991).

Studies have shown that the initiation and metastasis of gastric cancer is largely related to the loss of E-cadherin expression. E-cadherin encoded by gene *CDH1* has genetic and epigenetic abnormalities in both germline and sporadic gastric cancer, mainly including gene expression level changes, germline and somatic mutations, 16q22.1 allele deletion, promoter methylation andnoncoding RNA (noncoding RNA, ncRNAs) regulated epigenetic gene silencing, etc., leading to abnormal expression of E-cadherin. This paper aims to summarize the relationship between E-cadherin and the tumorigenesis, development, metastasis and drug resistance of gastric cancer from the aspects of biological function, inactivation mechanism and clinical significance, discussing the clinical application of E-cadherin in the early diagnosis, prognosis, and therapy of gastric cancer as well as development status of E-cadherin activator, which could provide new ideas for precise therapy of gastric cancer.

## E-Cadherin

### The Function of E-Cadherin

Cadherin which is a type of cell surface transmembrane glycoprotein has an important influence on the cell-cell adhesion function. E-cadherin belongs to class I classical cadherin, and it is an important part of the intercellular adhesion connection in epithelial tissue (Mendonsa et al., 2018).

Human E-cadherin is encoded by the *CDH1* gene and located on chromosome 16q22.1. The *CDH1* mutation is the only germline molecular mutation related to hereditary diffuse gastric cancer and lobular breast cancer (Gall and Frampton, 2013; Biswas, 2020; Blair et al., 2020). E-cadherin is a transmembrane glycoprotein consisting of three domains: extracellular domains (ECD), transmembrane domains and intracellular domains (ICD). ECD is composed of five cadherin repeats and contains 4 calcium ion binding sites, which mediate the adhesion function of E-cadherin; ICD interacts with α-, β-catenin and other catenin family members. It binds and connects to the cytoskeleton of actin to maintain the stability of cell structure, inhibiting the movement of individual cells, and participating in cell signal transduction (Gall and Frampton, 2013; Biswas, 2020). The abnormal expression of E-cadherin has a significant impact on the interaction between cells, leading to the destruction of the dynamic balance of epithelial tissues, making it easier for cells to gain mobility and invasiveness, making tumors more prone to infiltration and metastasis (Bruner and Derksen, 2018; Na et al., 2020).

### Roles of E-Cadherin in Gastric Cancer

#### The Expression of E-Cadherin in Gastric Cancer

Lots of studies have showed that E-cadherin is a critical tumor suppressor in several carcinomas, including GC (Birchmeier, 1995; Shimada et al., 2012). The decreased expression of E-cadherin was mainly found in diffuse type gastric cancer (Gamboa-Dominguez et al., 2005). 33–50% of sporadic diffuse gastric cancers have somatically inactive E-cadherin mutations. One of the articles depicted 63.6% of signet ring cell carcinoma patients were confirmed E-cadherin mutations (Machado et al., 2001). E-cadherin dysfunction may be contributed by lots of molecular mechanisms, including *CDH1* mutations (Oliveira et al., 2004), DNA hypermethylation (Grady et al., 2000; Machado et al., 2001), loss of heterozygosity (LOH), and non-coding RNAs that regulate E-cadherin expression. Among them, *CDH1* germline mutation is the main cause of diffuse gastric cancer.

And the expression of E-cadherin is an independent factor for the prognosis of gastric cancer. The previous study found that E-cadherin positive patients have better prognosis than that of negative patients (Wijnhoven et al., 2002). In addition, it has been reported in the literature that the prognosis of young patients is worse than that of the elderly (Schildberg et al., 2014).

#### Role of E-Cadherin in Tumorigenesis

E-cadherin plays an important role in the process of tumorigenesis. It can modulate intracellular signaling to promote tumor growth. Cell adhesion which E-cadherin mediateted, plays a central role in the Wnt signaling pathway. Under physiological conditions, β-catenin in the cytoplasm remains inactive by binding to the APC/GSK3β/Axin/CK1 degradation complex. Wnt signaling inhibits the degradation process by inhibiting the GSK3β complex through phosphorylation (Kourtidis et al., 2013; Zhan et al., 2017; Bruner and Derksen, 2018).The complex of the combination of E-cadherin and β-catenin has a negative regulatory effect on the β-catenin/Wnt pathway. Bruner HC et al. found that the increased density of extracellular matrix could destroy the E-cadherin/β-catenin complex of gastric cancer cells, thereby regulating the proliferation and the response to chemotherapy of gastric cancer (Jang et al., 2018). Moreover, Notch signaling is a critical way in tumorigenesis. It can induce the cyclooxygenase-2 (COX-2) expression through the binding of the Notch1 receptor intracellular domain to the COX-2 promoter, leading to tumor progression (Yeh et al., 2009). And inhibiting Notch signaling could induce G2/M cell cycle arrest through activating nuclear PTEN in gastric cancer (Kim et al., 2016).

In addition, changes of E-cadherin could cause the destruction of cell adhesion, leading to changes in loss of contact inhibition, and changes in cell migration and matrix interactions, thereby resulting in tumorigenesis (Berx and van Roy, 2009). E-cadherin deletion is regulated by a variety of factors, including changes in gene and mRNA levels. For example, *CDH1* germline mutations are closely related to the occurrence of hereditary diffuse gastric cancer (Guilford et al., 1998; Oliveira et al., 2004). In addition, the promoter DNA hypermethylation region and E-cadherin repressors (Machado et al., 2001), such as Snail, ZEB2/SIP1, and Slug, can combine with the E-box region of E-cadherin to induce E-cadherin inactivation, thereby promoting tumorigenesis (Batlle et al., 2000; Cano et al., 2000; Comijn et al., 2001; Conacci-Sorrell et al., 2003). Therefore, we will introduce these four inactivating mechanisms in E-Cadherin clearly. A better understanding of E-cadherin inactivation would provide an opportunity for future therapeutic intervention.

#### Gene Mutation

Loss of E-cadherin expression is closely related to the sporadic and genetic forms of gastric cancer, and *CDH1* mutation is one of the important reasons for the loss of E-cadherin expression (Corso et al., 2013). *CDH1* mutations include germline mutations and somatic mutations. Germline mutations can affect the entire coding sequence, including small frame divisions, splice sites, meaningless, missense mutations, and large rearrangements. According to reports, compared with missense mutations, non-missense mutations may be pathogenic, and the frequency of missense mutations is higher than that of nonsense mutations (Corso et al., 2011; Simões-Correia et al., 2012; Choi et al., 2020). High-frequency mutations at multiple sites in *CDH1* are one of the risk factors and signs of hereditary diffuse gastric cancer. And about 50–80% of diffuse gastric cancers have decreased or missing E-cadherin expression. Diffuse gastric cancer with *CDH1* mutation has more aggressive phenotypic characteristics, especially with a higher Ki67 marker index, p53 mutation and Her-2 positive gastric cancer (Muzashvili et al., 2020). And the clinical trail which is called “Hereditary Gastric Cancer Syndromes: An Integrated Genomic and Clinicopathologic Study of the Predisposition to Gastric Cancer” is recruiting.

*CDH1* gene mutation or transcription silencing is related to familial diffuse gastric cancer. Research confirms that *CDH1* mutations are more likely to be found in countries with a low incidence of gastric cancer.Thus preventive genetic screening is very important (Corso et al., 2021). And due to the poor sensitivity of gastroscopy for the detection of signet ring cell carcinoma, the monitoring of *CDH1* mutation patients is very limited. Therefore, prophylactic total gastrectomy may be the most desirable option for individuals with *CDH1* mutations and a family history of diffuse gastric cancer (Shenoy, 2019; Castro et al., 2020).

In addition to these inactivating *CDH1* mutation, the occurrence of gastric cancer is related to the polymorphisms of several single nucleic acid glycosides in the *CDH1* gene. Among them, the relation of *CDH1*-160C >a polymorphism to GC is currently the most extensively studied. In the present study, it found that the *CDH1*-160 AA genotype could increase the risk of gastric cancer (Zhang et al., 2008; Al-Moundhri et al., 2010).

#### DNA Methylation

DNA methylation is the addition of methyl groups at C5 of the cytosine ring to produce 5-methylcytosine. DNA methylation can control gene expression by changing chromatin structure, DNA conformation, DNA stability, and the way that DNA interacts with proteins. For example, the methylation of CpG islands in tumor suppressor gene promoters causes them to prevent transcription factors from entering the binding site in the promoter through steric hindrance, thereby inhibiting gene transcription. Promoter hypermethylation is considered to be another major mechanism for *CDH1* inactivation in the development of various cancers, including gastric cancer (Qu et al., 2013). And 79% of diffuse gastric cancer has found the promoter CpG hypermethylation, but only 55% of intestinal gastric cancer found it (Yoshiura et al., 1995; Ushijima and Sasako, 2004). Thus, according to the well-known two-hit inactivation mechanism proposed by Knudson, hypermethylation of the promoter CpG could be the second hit in abrogating E-cadherin expression (Carbone and Minna, 1993). IL-1b is demonstrated to be an important step in mediating E-cadherin methylation in *Helicobacter pylori*-related gastric cancer (Qian et al., 2008).

DNA methyltransferase 3A isoform B (DNMT3Ab) has been found to play a crucial role in the EMT process of gastric cancer. DNMT3Ab mediates epigenetic inactivation of E-cadherin gene through DNA hypermethylation and histone modification of H3K9me2 and H3K27me3. DNMT3Ab deletion effectively restored the expression of E-cadherin by reducing the methylation, H3K9me2, and H3K27me3 levels on the e-cadherin promoter. These results confirm that the DNMT3Ab targeting the DNMT3Ab/Snail/E-cadherin axis may provide a promising therapeutic strategy for the treatment of metastatic gastric cancer with high expression of DNMT3Ab (Cui et al., 2018).

#### Non-coding RNA

At present, more and more evidences show that non-coding RNA, including micro RAN (microRNA, miRNA) and long non-coding RNA (long non-coding RNA, lncRNA), play a vital role in carcinogenesis. So far, people have found a total of 2654 miRNAs described in the human genome (Kozomara et al., 2019). It is reported that miRNA can regulate the signal pathways related to E-cadherin, and inhibit the occurrence and development of gastric cancer by inhibiting the EMT process. Therefore, it is considered as a potential clinical biomarker for the treatment of gastric cancer (Lazar et al., 2008). For example, the increase of miR-1275 expression regulates the expression of vimentin/E-cadherin by directly inhibiting the expression of JAZF1, thereby inhibiting the metastasis of gastric cancer (Mei et al., 2019). It is reported that the expression of miRNA-203 is reduced in gastric cancer. Inhibition of miR-203 expression will increase the expression of phospho-ERK1/2 (pERK1/2) and Slug, as well as reduce the expression of E-cadherin, thereby promoting tumorigenesis (Gao et al., 2017a). Studies have shown that miR-21 inhibitors can inhibit tumor growth by up-regulating the expression of E-cadherin and PTEN, and down-regulating the expression of N-cadherin, β-catenin, vimentin, and Slug, suggesting that miR-21 may increase the expression of β-catenin. Promote endoderm transformation of gastric cancer cells induced by transforming growth factor PTEN1 (Li et al., 2016). MiR-340 targets SPP1 to inhibit the PI3K/AKT pathway, and activates the Snail/Slug signaling pathway, further inhibiting the proliferation, migration, invasion and EMT of gastric cancer cells (Song et al., 2019a; Chen et al., 2019). MiR-19a/miR-96-mediated low expression of KIF26A suppresses metastasis by regulating FAK pathway in gastric cancer (Ma et al., 2021). In intestinal gastric cancer, the loss of microRNA-101 can lead to E-cadherin functional downregulation through EZH2 up-regulation (Carvalho et al., 2012).

LncRNA is the main non-coding RNA complex with chromatin-modifying proteins. Previous studies indicated that lncRNA participates in the occurrence and progression of gastric cancer through regulating gene expressions at epigenetic, transcriptional or post-transcriptional levels. Therefore, they are considered as potential diagnostic hallmarkers (Yang et al., 2015; Yao et al., 2017). HOX antisense intergenic RNA (HOTAIR) is one of the earlistlncRNAs, and it is still one of the most widely studied. HOTAIR has been found to be upregulated in gastric cancer, and can promote tumor metastasis by binding to EZH2 with the E-cadherin promoter (Chen et al., 2017; Song et al., 2019b). Studies have found that HOTAIR and its combination with PRC2 inhibited the expression of miR34a, which controls the target genes C-Met (HGF/C-Met/Snail pathway) and Snail, thereby helping gastric cancer cells to develop EMT and promote tumor metastasis (Liu et al., 2015). Thus, the expression of HOTAIR was seemed to be an independent predictor of overall survival (Chen et al., 2017).The role of lncRNA CHRF promotes cell invasion and migration through EMT, which may provide a potential target for the biological diagnosis and therapy of gastric cancer (Gong et al., 2020). DCLK1, miR-15b, and lncRNA SNHG1 play a potential role in the occurrence of gastric cancer. Overexpression of lncRNA SNHG1 can promote the expression of DCLK1 and Nothc1 in gastric cancer cells. MiR-15b targets DCLK1 to regulate the expression of Notch1 and inhibit the EMT process of gastric cancer cells. LncRNA SNHG1 enhances the role of DCLK1/Notch1 in the EMT process by regulating the expression of miR-15b (Liu et al., 2020). LncRNA CCAT2 promotes gastric cancer proliferation and invasion through regulating the E-cadherin and LATS2 (Wang et al., 2016).And LINC00240 promotes GC tumorigenesis *via* a LINC00240/miR-124-3p/DNMT3B axis as an oncogene, so it may be a potential diagnostic biomarker for GC (Lazar et al., 2008).

### Role of E-Cadherin in Metasitasis

Metastasis is one of the key molecular steps affecting the prognosis of advanced GC patients. And Epithelial-mesenchymal transition (EMT) is one of the important steps in the process related to metastasis in gastric cancer (Valastyan and Weinberg, 2011).EMT refers to the phenomenon that epithelial cells transform into mesenchymal cells under specific physiological and pathological condition (Aban et al., 2021). And it can down-regulate the epithelial markers E-cadherin, ZO-1, etc., which can inhibite the characteristics and behavior of epithelial cells. At the same time, the mesenchymal markers N-cadherin and vimentin are up-regulated, and then activating the mesenchymal characteristics of the cell to relax the cells from the basement membrane, which is more spindle-shaped and motility in phenotype, and at the same time obtains apoptosis resistance. Therefore, EMT is considered to be one of the main mechanisms that determine the spread of invasive and metastatic cancer cells (Nieto et al., 2016).

The decrease of E-cadherin expression on the cell membrane leads to weakening or disappearance of the interaction between cells and inhibit the activation of transcription factors (Snail, Slug, Twist and ZEB-1), resulting in EMT (Kalluri and Weinberg, 2009; Aban et al., 2021). Studies have found that Snail family transcription factors are strong repressors of E-cadherin gene transcription. Epithelial cells that ectopicly express Snail have a fibroblast phenotype, and are tumorigenic and invasive. Therefore, the loss of E-cadherin protein may be due to germline mutations observed in diffuse gastric cancer, or it may be due to the over-expression of transcription inhibitors (Snail, Slug, Twist, ZEB-1) in advanced cancers.This phenomenon is transcription silence (Cano et al., 2000). And some studies found that Snail, Slug and ZEB1 expression were related to tumor differentiation, lymph node metastasis and pathological staging (Uchikado et al., 2011; Chen et al., 2016; Okubo et al., 2017; Xue et al., 2019). And EMT can activate β-catenin signaling pathway to promote tumor metastasis in gastric cancer (Park et al., 2015). It is demonstrated that abnormal activation of the hedgehog (Hh) signaling pathway may be involved in inducing EMT in gastric cancer.And the levels of the Hh pathway marker Gli-1 were associated with levels of the Snail and E-cadherin.All three markers were related to depth of invasion, lymph node metastasis, and pTNM stage in gastric cancer (Wang et al., 2014).

In addition, there are some signaling pathways that promote tumor invasion and metastasis by regulating the expression of E-cadherin. The Rho A, Rac1, and Cdc42. play a critical role in mataining the cytoskeleton, increasing cell fluidity, and taking part in the phenotype of cell mesenchyme. Increase of the RhoA aactivity resulted in the cancer cell proliferation and cell cycle disorders. Previous studies have shown that E-cadherin missense mutations associated with diffuse gastric cancer can induce an increase in RhoA activity, resulting in the proliferation and movement of gastric cancer cells (Suriano et al., 2003). In addition, E-cadherin can directly activate the Rho GTPase pathway, and it can also activate Rho GTPase through EGFR (Bremm et al., 2008). A recent study found that RhoA Y42 mutation is associated with poor prognosis of gastric cancer. Y42C mutation of RhoA has a higher protein level, which can promote the proliferation and movement of gastric cancer cells.As for EGFR, its activation is associated with the E-cadherin expression. And E-cadherin’s downstream effector molecules related to RAS and RAF/MEK pathways and other tumor-forming pathways such as FAK/C-SRC and PI3K/AKT/mTOR pathways, thus contributing to tumor cell proliferation and metasitasis. In addition, the loss of E-cadherin can increase β-catenin into the nucleus, thereby inhibiting PTEN expression, and then participating in the promotion of tumorigenesis through the AKT/mTOR pathway (Bremm et al., 2008). MMPs alterations are also important to tumor invasion and metastasis in gastric cancer. For example, in gastric cancer, the MMP1 and MMP2 increased expression is related to the loss of E-cadherin expression, thereby, leading cancer proferliation and metastasis (Zhou et al., 2010).MMP-3 and MMP-7 have association with the development of *Helicobacter pylori*-related gastric cancer (Yang et al., 2018).

### Role of E-Cadherin in Drug Resistance

Previous studies indicated that EMT played a critical role in drug resistance (Singh and Settleman, 2010; Chen et al., 2014; Lee et al., 2014). And the loss of E-cadherin expression is a key step in the EMT process. E-cadherin is significantly down-regulated or absent in tumor drug-resistant cells, which can increase the sensitivity of tumor cells to anti-tumor drugs (Selga et al., 2008; Gao et al., 2017b). E-cadherin can reverse the resistance of transformation therapy drugs by negatively regulating the expression of BCL-2, and up-regulate the expression of cell cycle inhibitor P27, exerting a proliferation inhibitory effect. It can also inhibit tumor cells by increasing the expression of tumor suppressor gene PTEN protein (Yang et al., 2008). The latest research data show that microRNA-421 regulated by hypoxia-inducible factor-1α promotes gastric cancer metastasis by targeting E-cadherin and caspase-3, inhibits cell apoptosis, and induces cisplatin resistance (Ge et al., 2016). Eukaryotic translation initiation factor 5A2 (EIF5A2) (by up-regulating epithelial markers E-cadherin and β-cadherin, down-regulating mesenchymal markers Vimentin and N-cadherin mediate the EMT process to regulate the resistance of gastric cancer cells to cisplatin (Sun et al., 2018).

### E-Cadherin and Helicobacter Pylori-Related Gastric Cancer

*Helicobacter pylori* (*Helicobacter pylori*, *H.pylori*) is one of the most common pathogens in humans. *H.pylori* infection can dysregulate Wnt signaling pathway and cause EMT in gastric tissue, then, increase the risk of gastric cancer. It induces a variety of inflammatory reactions by infiltrating macrophages, neutrophils, regulatory T cells and natural killer cells, which significantly affects the gastric microenvironment (Baj et al., 2020). *H. pylori* infection Cytoxin-associated gene A (CagA) is a HP virulence factor. Researc5h data found that *Helicobacter pylori* can induce epithelial-mesenchymal transition of gastric epithelial cells through CagA, thus showing characteristics similar to tumor stem cells (Bessède et al., 2014). VacA, another *H. pylori* virulence factor, could activate PI3K/Akt pathway and then induce Wnt/β-catenin signaling, leading to phosphorylation of GSK3β and translocation of the β-catenin to the nucleus to activate CCND1 gene (Nakayama et al., 2009). *H.pylori* infection can reduce the function of E-cadherin by activating the matrix MMP-3 and -7, and induce the migration and invasion of gastric cancer cells. It can also regulate miRNA, such as miR-128/miR-148a, and affect the MMPs/E-cadherin signaling pathway, thereby promoting the occurrence of tumors (Yang et al., 2018).In addition, *H.pylori* could initiate E-cadherin methylation which may subsequently progress to intestinal metaplasia and invasive cancer.But it need to be further investigated (Chan et al., 2003a; Qian et al., 2008).

Nuclear factor kappa B (NF-κB) signaling way is the most important way in *H.pylori* -related gastric cancer (DiDonato et al., 2012). NF-κB is a group of transcription factors (RelA, RelB, c-Rel, NF-κB1/p50, and NF-κB2/p52), which form homo- and heterodimers and upregulate or suppress expression of many genes (Neumann and Naumann, 2007). The NF-κB-driven gene products include cytokines/chemokines IL-1, IL-8, TNF, IL-6, MCP-1, pro- ,and anti-apoptotic factors cIAPs, c-FLIP, A20, Bcl-XL, angiogenesis regulator vascular endothelial growth factor (VEGF), matrix metalloproteinases (MMP)-2, MMP-9 in non-transformed or tumor cells in response to a variety of stimuli, including growth factors, cytokines, hormones, microbial and chemical compounds (Maeda and Omata, 2008). In a *H. pylori* infection gastric cell line, the infection can promote NF-κB- and AKT-mediated MMP-9 production, which lead to cell migration and invasion (Maubach et al., 2013). And previous study has demonstrated that NF-κB decreases the expression of E-cadherin in gastric cancer through regulation of other transcription factors, promoting EMT, tumor angiogenesis and metastatic dissemination (Nam et al., 2011; Hu et al., 2013).

## Clinical Significance of E-Cadherin

E-cadherin plays an important role in cell connection, and loss of E-cadherin is crucial in the occurrence and development of gastric cancer. Data show that E-cadherin deletion is often associated with poorly differentiated cancer, lymph node metastasis, and tumor staging (Gao et al., 2017b). Therefore, evaluating the protein level of E-cadherin and changes in the *CDH1* gene may provide promising prospects for the diagnosis, prognosis, and treatment of gastric cancer.

### E-Cadherin Can be Used as a Potential Marker for the Diagnosis of Gastric Cancer

Kahtan Al-Bayaty, Mustafa and others tested the serum E-cadherin status of 30 gastritis patients, 20 gastric ulcer patients, and 20 gastric cancer patients. They found that the E-cadherin level of all patients increased, and the gastric cancer group was significantly higher than the other groups. Increase, so E-cadherin can be used as a potential marker for the diagnosis of gastric cancer (Carneiro et al., 2012). The extracellular domain of E-cadherin can be broken down into 80 kDa fragments by proteases under certain pathological stimuli. These fragments can be soluble E-cadherin (sE-cadherin). In patients with intestinal and diffuse gastric cancer, serum soluble E-cadherin also presents a completely different pattern. The level of serum E-cadherin increases in intestinal gastric cancer, while in diffuse gastric cancer, especially in the advanced stage, its level decreases, so it may be a biomarker for the diagnosis of intestinal gastric cancer (Juhasz et al., 2003).

### E-Cadherin Can be Used as a Predictor of Gastric Cancer Prognosis

The abnormal expression of E-cadherin is related to the aggressiveness of gastric cancer, suggesting that this marker may serve as a negative prognostic factor for gastric cancer (Lazar et al., 2008; Xia et al., 2017; Kumar et al., 2021). Zhila Torabizadeh et al. detected the protein expression level of E-cadherin in the tumor tissues and adjacent tissues of 70 patients with gastric cancer. And they found among these 70 patients, 48.6% showed abnormal E-cadherin expression**.**the abnormal expression of E-cadherin has a strong correlation with tumor stage, tumor grade, depth of invasion and local lymph node involvement, this marker can be used as a predictor of tumor aggressiveness in gastric cancer (Torabizadeh et al., 2017; Wool Eom et al., 2020).

It can be seen that detecting the expression of E-cadherin or the alteration of the *CDH1* gene encoded by it may provide promising applications for the diagnosis, prognosis or treatment targets of gastric cancer. E-cadherin and β-cadherin are often more related to advanced disease and poor prognosis. Chan AO et al. measured the expression of soluble E-cadherin in 116 patients with gastric adenocarcinoma included in the trial, and found that soluble E-cadherin can be used as a long-term predictor of cancer patients. It is an independent factor of survival, and it can be a potentially valuable prognostic factor for patients with gastric cancer before treatment (Chan et al., 2003b).

## Development of E-Cadherin Activator

Although EMT is a complex process that is regulated by multiple genes, the loss of E-cadherin is one of the most important characteristics. Therefore, compounds that target to restore E-cadherin expression may become potential EMT reversal agents and provide new ideas for inhibiting tumor metastasis.

So far, many compounds have been found to restore E-cadherin expression. Here, as shown in Table 1, we summarize the compounds that can restore E-cadherin protein expression and inhibit EMT in gastric cancer. In the HDGC, hypermethylation of the promoter CpG could be the second hit in abrogating E-cadherin expression, therefore, the histone deacetylase (HDAC) inhibitors and DNA-demethylating agents has become potential drugs that can regulate expression of E-cadherin (Grady et al., 2000). HDAC inhibitors are able to block substrate recognition and inducing gene expression (Sharma et al., 2010). Oxamflatin, the HDAC inhibitors can induce E-cadherin expression and reduce cell viability in gastric cancer. Thus, it can be further considered for the prevention of tumor metastasis (Faghihloo et al., 2016). DNA methylation inhibitors are nucleoside analogs that can inhibit DNA methylation by trapping DNA methyltransferases, leading to their depletion inside the cell (Egger et al., 2004). Decitabine (DAC), a DNA methylation inhibitor, has been demonstrated to promote gastric cancer cell migration and invasion *via* the upregulation of NEDD4-1 (Li et al., 2015). Cyclooxygenase-2 (COX-2) participates in cancer invasion and metastasis by regulating E-cadherin expression through the NF-κB/Snail signaling pathway in gastric cancers (Chen et al., 2013; Liu et al., 2013). Thus COX-2 inhibitor have been shown to be chemopreventive against gastric cancer (Ohno et al., 2001; Hu et al., 2004). Celecoxib, a COX-2 selective inhibitor, has been demonstrated to induce apoptosis and inhibit angiogenesis of gastric cancer and then has an inhibitory impact on E-cadherin, resulting in suppressing the invasion of advanced gastric cancer (Zhou et al., 2007). And the potential application of Allium genus to GC chemoprevention and treatment support through *CDH1* restoration and COX2 downregulation. In addition, an estrogen derivative megestrol and other estrogen receptor modulators can specifically inhibit the viability of gastric cancer cells by inducing apoptosis before DNA damage (Shimada et al., 2018). The latest evidence shows that luteolin affects cell proliferation, migration, apoptosis and reverses EMT by inhibiting Notch1 pathway, thereby inhibiting the progression of gastric cancer (Zang et al., 2017). Metformin can inhibit EMT in a glucose-independent manner, thereby inhibiting the invasion and migration ability of gastric cancer cells (Valaee et al., 2017). The recently research discovered that, NEDD8-Activating Enzyme (NEDD8-Activating Enzyme, NAE) inhibitor MLN4924 can significantly inhibit the migration of gastric cancer cells through transcriptional activation of E-cadherin and inhibition of MMP-9 (Lan et al., 2016). In addition, the natural compound triallyl trisulfide (DATS) extracted from garlic can inhibit gastric cancer cell metastasis by up-regulating E-cadherin and down-regulating MMP-9 (Jiang et al., 2017). Dihydromyricetin (DHM) up-regulates E-cadherin and down-regulates Vimentin through the JNK/MMP-2 pathway, inhibiting the migration and invasion of gastric cancer cells (Wang et al., 2019). Icariin mesylate (Eribulin) can inhibit the EMT changes of triple-negative breast cancer. Kurata T et al. further found that in gastric cancer cells, it can inhibit EMT by regulating the TGF-β/Smad signaling pathway. Icariin sulfonate combined with 5-FU may be a promising treatment option for the treatment of gastric cancer peritoneal metastasis (Kurata et al., 2018). Astragaloside inhibits the conversion of E-cadherin to N-cadherin induced by TGF-β1 by inhibiting the PI3K/Akt/NF-κB pathway to inhibit the survival, invasion and metastasis of gastric cancer cells (Zhu and Wen, 2018). Li Nan et al. found that dihydroartemisinin can effectively inhibit the malignant proliferation of gastric cancer cells, down-regulate the activities of PI3K/AKT and Snail, and inhibit the epithelial-mesenchymal transition of gastric cancer cells (Li et al., 2019). And the research reveals that, the curcumin could inhibit the migration and invasion of gatric cancer cells, downregulate the expression of N-cadherin, snail1, Wnt3a, p-β-catenin, p-LRP6, and Bcl-2, and upregulate the expression of E-cadherin and Bax. Therefore, it might provide potential strategies for gastric cancer treatment (Liu et al., 2019).

**TABLE 1:** Compounds that restore E-cadherin protein expression in gastric cancer.

Drug	References	Characteristic	Function and mechanism
Astragaloside	Zhu and Wen, (2018)	A lanolin alcohol type tetracyclic triterpene saponins and one of the main active ingredients of Astragalus	Inhibit PI3K/Akt/NF-κB pathway and inhibit the conversion of E-cadherin to N-cadherin induced by TGF-β1
Celecoxib	Zhou et al. (2007)	Selective COX-2 inhibitor	Induce apoptosis a, inhibit angiogenesis, and increase the expression of
Cariin mesylate eribulin	Kurata et al. (2018)	Flavonoid glycosides derived from plants of the genus epimedium	Down-regulate the TGF-β/Smad signaling pathway
Curcumin	Liu et al. (2019)	A diketone compound extracted from plant rhizomes	Downregulate the expression of N-cadherin, snail1, Wnt3a, p-β-catenin, p-LRP6 and Bcl-2, and upregulate the expression of E-cadherin and bax, and increase the activity of caspase-3, caspase-8, caspase-9
Decitabine	Li et al. (2015)	DNA methylation inhibitor	Upregulation of NEDD4-1
Dihydroartemisinin	Li et al. (2019)	Artemisinin derivatives	Down-regulate the activities of PI3K/AKT and snail, and inhibit the epithelial-mesenchymal transition of gastric cancer cells
Dihydromyricetin (DHM)	Wang et al. (2019)	Traditional Chinese medicine with extensive anti-tumor effects	Up-regulate E-cadherin and down-regulates vimentin through the JNK/MMP-2 pathway
Estrogen derivative megestrol and other estrogen receptor modulators	Shimada et al. (2018)	Estrogen receptor modulator	Induce apoptosis before DNA damage
Luteolin	Zang et al. (2017)	A flavonoid found in fruits and green plants	Inhibite Notch1 signal transduction
Metformin	Valaee et al. (2017)	An anti-diabetic drug for treating type 2 diabetes	Decrease mesenchymal markers (including vimentin and β-catenin), and induce epithelial markers (E-cadherin)
MLN4924	Lan et al. (2016)	NEDD8-activating enzyme inhibitor	Activate E-cadherin and inhibit MMP-9
Oxamflatin	Faghihloo et al. (2016)	HDAC inhibitor	Induce E-cadherin expression and reduce cell viability
Triallyl trisulfide (DATS)	Jiang et al. (2017)	Garlic extraction	Increase the phosphorylation of cyclin A2, cyclin B1, JNK, ERK and p38, activate the MAPK pathway, up-regulate the expression of E-cadherin and down-regulate the expression of MMP-9

## Summary and Outlook

Although the current clinical therapy of gastric cancer has made great progress, its effect is far from satisfactory. In China, owing to lack of early symptoms obvious and biomarkers for early diagnosis, about 75% of gastric cancer have been at advanced stage when diagnosed, and usually accompanied by metastasis. Therefore, early diagnosis of gastric cancer is important to reduce mortality. E-cadherin maintains the integrity of the epithelial cell layer through cell adhesion. The decreased expression of E-cadherin in gastric cancer is related to the malignancy and poor prognosis of gastric cancer patients. The study found that soluble E-cadherin could be used as an independent factor to predict the long-term survival of tumor patients, and it could be used as a biomarker for the early diagnosis of gastric cancer, with broad application prospect. Because patients with advanced gastric cancer have poor therapeutic effect and poor sensitivity to radiotherapy, chemotherapy, immunotherapy, etc., precise targeted therapy is more important for the treatment of gastric cancer. In gastric cancer, diffuse gastric cancer is more closely related to E-cadherin expression and *CDH1* mutations. Diffuse gastric cancer with *CDH1* mutation has more aggressive phenotypic characteristics, and targeted E-cadherin therapy may provide new ideas for inhibiting tumor metastasis, which can be used as a potential target for future gene and genetic therapy and has important value.
